# Best of Intentions: Influential Factors in Infant Feeding Intent among Marshallese Pregnant Women

**DOI:** 10.3390/ijerph19031740

**Published:** 2022-02-03

**Authors:** Britni L. Ayers, Rachel S. Purvis, Alexis White, Sheena CarlLee, Jennifer A. Andersen, Cari A. Bogulski, Pearl A. McElfish

**Affiliations:** 1 College of Medicine, University of Arkansas for Medical Sciences Northwest, 1125 N. College Ave., Fayetteville, AR 72703, USA; rspurvis@uams.edu (R.S.P.); swcarllee@uams.edu (S.C.); jaandersen@uams.edu (J.A.A.); pamcelfish@uams.edu (P.A.M.); 2 Department of Obstetrics and Gynecology, University of Arkansas for Medical Sciences, 4301 W. Markham St., Little Rock, AR 72205, USA; acwhite@uams.edu; 3 Office of Community Health and Research, University of Arkansas for Medical Sciences Northwest, 1125 N. College Ave., Fayetteville, AR 72703, USA; cbogulski@uams.edu

**Keywords:** Marshallese, infant feeding intentions, infant nutrition, exclusive breastfeeding

## Abstract

The purpose of this study is to explore the beliefs, intentions, and influences that serve as barriers and facilitators to exclusive breastfeeding intent among Marshallese pregnant women in the United States (US). The study used a descriptive qualitative design. In total, 36 Marshallese women in their third trimester of pregnancy participated. Participants described exclusive breastfeeding as the preferred method of infant feeding, from both individual and community perspectives. Exclusive breastfeeding was viewed as the healthiest for the infant, viewed as offering protection against sickness, and viewed as better for the overall development of the infant. Of the 36 participants, 28 participants (77.8%) stated that their infant feeding intentions included a hybrid of breastfeeding and formula feeding. The dominant barrier to exclusive breastfeeding was the need to work outside of the home. Unexpected barriers to exclusive breastfeeding were the desire for autonomy and a preference to exclusively breastfeed female infants more than male infants. Exclusive breastfeeding facilitators included support from the Special Supplemental Nutrition Program for Women, Infants, and Children and support and encouragement from female family/community members. This study is the first to document beliefs, intentions, and influences that serve as barriers and facilitators to exclusive breastfeeding among Marshallese pregnant women residing in the US.

## 1. Introduction

The Centers for Disease Control and Prevention (CDC) [1] recommends exclusive breastfeeding (breast milk only) for infants in the first six months of life with the introduction of complementary feeding while maintaining breastfeeding up to two years. Although exclusive breastfeeding rates have increased overall in the United States (US), racial and/or ethnic minority inequities persist [2]. Breastfeeding behavior varies based on income, education, informal and formal support, and acculturation among migrant women once they reside in the US [3,4,5,6].

Pacific Islanders residing in the US have the lowest rates of exclusive breastfeeding initiation and duration [2]. Women of Pacific Islander heritage have the lowest rate of exclusive breastfeeding at six months (12%), compared with African American (23%), Hispanic (18%), and non-Hispanic Whites (23%) [7]. Only 12% of Pacific Islander infants in the US are exclusively breastfed at six months, compared with 31% of infants in the US Affiliated Pacific Islands and 25% for other populations in the US [7]. Pacific Islanders living in the US also experience disparities in multiple conditions known to be prevented or mitigated by exclusive breastfeeding practices, such as cardiovascular disease [8], cancer [9], diabetes [10,11], and cardiometabolic disease over the life course [12,13,14,15,16]. Exclusive breastfeeding has protective factors for cardiometabolic diseases for mother and infant. For infants, exclusive breastfeeding decreases the risk of obesity, sudden infant death syndrome, and acute conditions such as gastrointestinal and respiratory infections. In the longer term, exclusive breastfeeding can reduce the risk of childhood obesity [17,18,19]. For the mother, data suggest that exclusive breastfeeding may reduce the risk for breast and ovarian cancer, hypertension, hyperlipidemia, metabolic syndrome, and type 2 diabetes [20,21,22,23,24].

From 2000 to 2010, the Pacific Islander population in the US increased by 40%, growing three times faster than the total US population and making it the second-fastest-growing population in the US. The fastest growth occurred in the South (66%), especially in Arkansas (252%), where the majority of Pacific Islanders are Marshallese [25]. Arkansas has the largest population of Marshallese living in the continental US (~14,000 people) [26,27,28,29,30]. Marshallese culture is matriarchal and collectivist, with immense value placed on motherhood [31,32,33,34,35,36]. Now that the Marshallese community has migrated to the US, their maternal culture has been disrupted, affecting reproductive life planning, pregnancy, birth, and especially their infant feeding behavior and experiences [31,32,33,34,35,36,37]. However, due to the aggregation of Pacific Islanders with other Asian groups, there are little-to-no statistics on health behaviors such as exclusive breastfeeding for Pacific Islander subgroups such as the Marshallese [38,39,40,41]. Of the limited literature, one study has reported very low breastfeeding rates among Marshallese Pacific Islanders, with a precipitous decline in exclusive breastfeeding after migration to the US [42,43]. Further, qualitative literature identifies that although Marshallese mothers view exclusive breastfeeding as the healthiest option for their infant, they are experiencing new challenges since after migrating to the US, including verbal and non-verbal shaming of breastfeeding while in public, perceived poor milk supply and quality, and the inability to breastfeed after they return to work [37].

Currently, there is no research to explore exclusive breastfeeding intentions among Marshallese pregnant women. Research demonstrates that exclusive breastfeeding intentions are stronger predictors of exclusive breastfeeding behavior than socio-demographic characteristics [44]. The purpose of this study is to explore the beliefs, intentions, and influences that serve as barriers and/or facilitators to exclusive breastfeeding behavior among Marshallese pregnant women residing in the US.

## 2. Methods

### 2.1. Study Design

A descriptive qualitative design was used as an exploratory method to better understand beliefs, intentions, and influences related to exclusive breastfeeding among Marshallese living in northwest Arkansas. Participants also completed a brief demographic survey (Supplementary Materials). A community-based, participatory research (CBPR) approach was used to design and implement the study. CBPR is an approach that honors and integrates Marshallese cultural values and practices into every aspect of the research [45,46,47]. To ensure cultural appropriateness, this study was guided by the Healthy Start Community Action Network (CAN) advisory board of local Marshallese community members and health care professionals. Several members of the interprofessional research team are Marshallese. All study plans and documents, including those used for recruitment, consent, and retention, as well as the quantitative surveys and qualitative interview guides, were developed with intensive input from the CAN and Marshallese study staff [36]. This study was conducted according to the guidelines laid down in the Declaration of Helsinki, and all the procedures involving research study participants were approved on 30 March 2018 by the University of Arkansas for Medical Sciences Institutional Review Board (#217929).

### 2.2. Participant Eligibility, Recruitment, and Consent

Women were eligible to participate in the study if they (1) self-identified as Marshallese; (2) were 18 years of age or older; and (3) were between 28 and 39 weeks gestation to assess exclusive breastfeeding intention. Exclusion criteria included (1) conception with the use of fertility treatments; (2) multiple gestations (pregnant with more than one infant); and (3) use of medications known to influence fetal growth (e.g., glucocorticoids, insulin, thyroid, hormones, etc.) since these components would classify participants as a higher-risk group. Marshallese bilingual study staff recruited women in their third trimester of pregnancy from local clinics, faith-based organizations, and community-based organizations [32,37]. Potential participants who met the inclusion criteria were offered the opportunity to join the study and complete the consent process. All participants approached met the inclusion criteria, for a 100% participation rate. Trained, bilingual female Marshallese study staff conducted the consent process. Study staff read the consent aloud to the participant in the participant’s language of choice (English or Marshallese). Participants had the opportunity to ask questions and have their questions answered prior to consent. Participants were provided a copy of the consent in English and Marshallese.

### 2.3. Sample Size and Saturation

A recruitment goal of 30 participants was chosen because that has allowed for saturation in other qualitative research with Marshallese women about breastfeeding behavior [37]. Saturation occurs when redundancy is reached in data analysis and signals to researchers that data collection may cease [48]. While we reached saturation at 30 participants, we continued to enroll until we reached 36 participants, which allowed for more nuanced responses. There are approximately 300 Marshallese infants born annually in Arkansas, making 36 participants an appropriate and representative sample [49].

### 2.4. Instrument Development and Data Collection

Data were collected from July 2019 to July 2020. The CAN and bilingual Marshallese study staff co-developed, reviewed, edited, and approved the quantitative survey and qualitative interview guide prior to data collection. The quantitative survey underwent three iterations to ensure cultural appropriateness. The quantitative survey was implemented using Research Electronic Data Capture (REDCap) [50] and took approximately 15–30 min to complete. The qualitative interview guide went through five iterations to ensure cultural appropriateness. Qualitative interviews lasted approximately 30 min to an hour. Marshallese bilingual study staff translated the instruments into Marshallese. After translations, the study team including Marshallese bilingual study staff conducted six mock data collection events over the course of four months. These mock data collection events served as training for the Marshallese bilingual study staff and allowed the team to evaluate any challenges in cultural nuance, comprehension, and translations of study materials. All data were captured by the trained, bilingual Marshallese study staff and occurred at a non-profit organization in Springdale, which has the highest Marshallese population in the region. Participants received a 40 USD Walmart gift card after completing the data collection event.

### 2.5. Data Analysis

Descriptive statistics, including means and standard deviations for continuous variables and proportions for categorical variables, were tabulated and presented to characterize participants’ demographics and summarize the exclusive breastfeeding intentions of participants. Interviews were audio recorded and transcribed verbatim in the language spoken by participants. Any transcripts in Marshallese were translated into English. Three trained researchers in qualitative analyses began with initial coding, which consists of naming each data segment with short summations. This process helped organize the data for focused codes. The focused codes that emerged were used to identify and develop the most salient categories within the data [51]. The research team discussed the emergent themes to ensure scientific rigor and inter-coder agreement. There were two primary coders and one confirmation coder. The qualitative analytic approach integrated inductive and deductive techniques. Member checks with Marshallese bilingual study staff were implemented. Member checking is a validation technique to discuss the credibility of results and is critical in CBPR research [48]. Marshallese have a collectivist culture, and it is common and expected for a family member, most often an adult child, to speak on behalf of the patient. In addition, many Marshallese within one family will use inclusive language such as “we” or speak in the third person to include those in their family and community in the statement, as some quotes may reflect [36,52].

## 3. Results

### 3.1. Participant Characteristics

Table 1 shows participants’ demographic characteristics (*n* = 36). Participants’ mean age was 27.1 ± 6.1 years, and 88.9% were born in the Marshall Islands. A majority of the participants were single, widowed, or in an unmarried partnership (77.8%). Most of the participants had a high school education or lower (83.3%) and were unemployed (75%). Household size ranged from 4 to 10. A little less than half of the participants had no health insurance (38.9%). Participants’ mean number of pregnancies was 3.4 ± 1.9. Over half of the participants had their first prenatal appointment in their second or third trimester (52.7%) rather than the first trimester. A majority of the participants’ infant feeding intentions included both breastfeeding and formula feeding (77.8%), with 13.9% intending to exclusively breastfeed and 8.3% intending to exclusively formula feed.

### 3.2. Qualitative Results

Table 2 includes primary themes and the subthemes. Three primary themes emerged: (1) Breastfeeding Beliefs and Intentions; (2) Breastfeeding Barriers; and (3) Breastfeeding Facilitators.

#### 3.2.1. Breastfeeding Beliefs and Intentions

Participants discussed their breastfeeding beliefs and intentions during their third trimester of pregnancy. Within this theme, three subthemes emerged: (1) Individual Breastfeeding Beliefs; (2) Marshallese Community Breastfeeding Beliefs; and (3) Infant Feeding Intentions (Table 2). Representative quotes are labeled by packet identifier (PID) number only to maintain anonymity.

##### Individual Breastfeeding Beliefs

The majority of the participants described breastfeeding as the best and healthiest option for the infant and preferred it over formula feeding. A participant explained, “When they breastfeed, their babies are healthier than when they are bottle-fed” (PID 22). Participants consistently echoed this sentiment with statements such as “They get more vitamins from their mom’s breast milk than formula. The ones that breastfeed have healthy babies” (PID 5) and “I think it’s good because breastfeeding is healthier” (PID 2).

In addition to the belief that breastfeeding was the healthiest option, there was also a belief that breastfeeding promoted better overall development for the infant. One participant said, “The ones that exclusively breastfeed, I heard that it’s good because they grow up and develop better with breast milk than formula” (PID 21). Another participant agreed and discussed how breastfeeding helps with healthy child development when she said, “I think it is a good thing to breastfeed because it is healthy for the baby. It helps the brain” (PID 36).

Participants also discussed how breastfeeding aids in building immunity against sickness for infants. One participant said, “I think it’s smart because babies with bottles, if they don’t have medicine, they cannot use their mom’s milk as a shield so I think that’s the main reason I like breastfeeding too” (PID 19). Another participant said:

“One of my girlfriends said breastfeeding is better and she breastfed all her kids and so it’s healthier and they don’t get sick compared to bottle feeding (formula feeding). If there is a flu going around this week or this month they won’t get it”.

(PID 34)

Another participant agreed and stated, “The ones that exclusively breastfeed their babies are protecting their babies from getting sick, so that they don’t get sick easily. And those that bottle feed their babies the babies are likely to [get] sick easily” (PID 14). Breastfeeding was described as a safeguard against sickness, and breastmilk was described as a healing agent topically. One participant said:

“I think breastfeeding is good because the breast milk is good. Aside from breastmilk being better and more nutritious for the baby, if they have insect bites, the breast milk is also good for that. You can put some breastmilk on the bite and it heals it”.

(PID 35)

##### Marshallese Community Breastfeeding Beliefs

Participants were asked about the Marshallese community’s beliefs about breastfeeding. The majority of the participants described the community as preferring breastfeeding over formula feeding. Much of the discourse identified in the participants’ individual views of breastfeeding mirrored that of the community beliefs. One participant said, “They truly believe in breastfeeding, it’s like their number one method” (PID 5). Another participant said, “I feel like most Marshallese women or ladies or girls breastfeed their kids” (PID 30).

Participants consistently described identical beliefs within the community as their own when they said, “They think it’s smarter. It’s the best decision for your kid’s health” (PID 19); “They say that breastfeeding makes the baby healthier” (PID 22); and “They think it’s good so the babies grow faster and healthy and not get sick easily” (PID 5).

##### Infant Feeding Intentions

Although all of the participants preferred exclusive breastfeeding over bottle feeding, almost all of the participants stated that their infant feeding intentions included both breastfeeding and bottle feeding (77.8%) (Table 1). When asked about their infant feeding intentions, participants stated, “I will do both breast and bottle feed” (PID 15); “I will do both breast and bottle feeding (formula feeding)” (PID 14); “I will do both” (PID 16); “I will do both” (PID 25); and “Both breastfeed and bottle” (PID 4).

Participants discussed breastfeeding and bottle feeding until it was time to introduce foods. Participants said, “Both, until it’s time to introduce solid foods” (PID 21) and “I will do both breast and bottle feeding (formula feeding) until it’s time for solids” (PID 24).

Only three participants stated that they intended on exclusively breastfeeding their infants. Two participants said that they intended to bottle feed only.

#### 3.2.2. Breastfeeding Barriers

Participants described the most influential barriers to their breastfeeding intention decisions. Within this theme, three subthemes emerged: (1) Work; (2) Independence/Autonomy; and (3) Gender (Table 2).

##### Work

The strongest barrier for the intention to exclusive breastfeeding that emerged was the need to return to work. Embedded in this discourse was the belief that if they planned to return to work, they had no choice but to bottle feed. For example, one participant stated:

“I see a lot of them bottle-feed, like most of the people I know bottle feed their babies because they work and they leave their babies with babysitters and so they bottle feed them. The way I see it all depends on work. As we all have to work, us Marshallese, so if you work you have no choice but to bottle-feed your kid”.

(PID 28)

Participants’ infant feeding intention to accomplish both tasks was mirrored in their qualitative responses and was based on their need to return to work. Participants’ responses were highly similar, and identical sometimes, with statements such as “I will do both because I’m planning to work” (PID 14); “I will do both because I am planning to work” (PID 24); “I am going to do both because I am planning to go back to work” (PID 27); “I plan to alternate so I can work” (PID 28); and “I want to work, he will bottle-feed while I’m at work and breastfeed when I am at home” (PID 5).

##### Independence/Autonomy

Another barrier that helped explain participants’ infant feeding intentions was the desire for autonomy and overall independence from the infant. One participant explained why she wanted to use formula feeding:

“It makes the baby able to be independent from the mom when she needs to go somewhere. The baby will be able to stay home and not cry when mom is at work. The kids don’t want to let go. The mom lays down for breastfeeding their babies and that makes the babies more attached and cling more to them”.

(PID 24)

Participants described a desire for independence. One participant said, “I will probably breastfeed for six months. It all depends so she doesn’t get too attached to me” (PID 32). Another participant shared this sentiment and stated, “I don’t like the fact that the baby is always clinging to me” (PID 41).

The infant feeding intention to formula feed was described as facilitating autonomy. One participant said, “They have their grandmothers babysit while they go out and about with their friends” (PID 31). Despite the ubiquitous belief that exclusive breastfeeding is best for the infant, there was discussion of personal choice. One participant said, “I think that everybody that does that is doing a good thing but if they don’t do it, it’s their choice” (PID 30).

##### Gender

A unique subtheme that emerged as a barrier to exclusive breastfeeding was the predilection to exclusively breastfeed females more than males. Some participants described this infant feeding choice as gender led. For example, one participant said, “She [referring to her daughter] coughs and doesn’t like the formula but my boys had formula” (PID 14). Another participant said, “I will try to breastfeed my baby. I will try. I will do both. If he doesn’t like the breast then I will use the bottle to feed him” (PID 1). Another participant discussed exclusively breastfeeding her daughter but not her sons when she said, “All my boys didn’t like my breastmilk and I had to change from breastfeeding to formula, they didn’t breastfeed for long and they didn’t like it” (PID 4).

Although the qualitative responses do not explain the gender divide completely, one participant stated that this infant feeding decision was situated in culturally specific gender roles when she said:

“Well, my daughter, it took her three years. It was super hard for her to let go. So I said, maybe give her a couple more months and I was breastfeeding both of them. I think it’s easier to stop my son because he is a boy and it’s ok if he cries. It is more different for you if you’re having a girl than a boy”.

(PID 19)

#### 3.2.3. Breastfeeding Facilitators

Participants described the influences that facilitated their exclusive breastfeeding intention decisions. Within this theme, two subthemes emerged: (1) Institutional Facilitator and (2) Matriarchal Facilitator.

##### Institutional Facilitator

The dominant institutional facilitator for breastfeeding that emerged among the participants was the overarching support from the Special Supplemental Nutrition Program for Women, Infants, and Children (WIC). A majority of the participants reported they were not enrolled in WIC (63.9%). However, qualitatively, the participants who were enrolled described WIC as supportive of exclusive breastfeeding. One participant said, “I was at the WIC office and they recommended exclusive breastfeeding for my baby” (PID 14). Another participant described how WIC and her health care providers were supportive in encouraging breastfeeding when she said, “WIC, and even my nurses. Like my doctors even asked what I am going to do and if I say just bottle-feed, they said how about think of both, to be better” (PID 19).

The majority of the participants said that WIC provided breastfeeding education specifically. One participant said, “WIC, they provide and help educate us on how to feed our babies” (PID 2). Another participant said, “WIC, they help provide education on breastfeeding and what’s good for breastfeeding moms” (PID 5). Another participant said:

“The folks at WIC educate us on how to measure the formula and how much water to use. They say that the breast milk has more vitamins than the formula so it is best to breastfeed and will help prevent the baby from getting sick”.

(PID 22)

##### Matriarchal Facilitator

Participants identified matriarchal family support as a strong influence for breastfeeding. One participant said, “I learn and listen to my mom mostly. My mom and grandma teaches me and they remind me all the time on how to feed my babies” (PID 24). Another participant said, “My mom recommends that I breastfeed” (PID 28).

The maternal support was focused on the health of the infant using a cultural narrative. For example, one participant said:

“My mom always told me to breastfeed. My mom says it’s healthy. She said because they’re eating from the family, I don’t know how she explained it to me but she used [too] big of Marshallese words so I don’t know”.

(PID 34)

Another participant said, “Maybe because I’ve been around my mom and heard her tell me to breastfeed my babies so that they don’t get sick easily” (PID 24).

## 4. Discussion

Participants described exclusive breastfeeding as the preferred method of infant feeding, from both individual and community perspectives. Participants believed exclusive breastfeeding was the healthiest for the infant, offered protection against sickness, and was better for the overall development of the infant. The previous literature evaluating exclusive breastfeeding beliefs among Marshallese women identified that the majority of Marshallese women viewed breastmilk as superior to formula [37]. Although this finding was anticipated, previous analyses for Marshallese mothers’ exclusive breastfeeding beliefs were based on postpartum recall and did not identify perspectives of pregnant Marshallese mothers suggesting the belief that exclusive breastfeeding is the preferred method at both pre- and postpartum.

While all participants discussed breastfeeding as the healthiest option, most of the participants (77.8%) (Table 1) stated that their infant feeding intentions included a hybrid of breastfeeding and formula feeding. The participants described the dominant barrier to their intent to exclusively breastfeed as the need to work outside of the home. Returning to work is a commonly identified motivator to not exclusively breastfeed and has been previously identified among other minority women and Marshallese women [37,53,54,55]. However, this study identified that participants felt they had *no choice* but to formula feed, potentially suggesting a lack of supportive resources to maintain exclusive breastfeeding while working outside of the home for this specific population. The majority of the participants (75%) were unemployed, with 83.3% having a high school education or lower. Research shows that exclusive breastfeeding is not an option for the majority of poor, less educated, and non-professional working women, due to the lack of formal and informal support within non-professional working environments [56,57,58]. The state of Arkansas is ranked second to last, with an overall exclusive breastfeeding rate of 19.4%, which may be attributable in part to the state’s low socio-economic and educational status [59]. Moreover, due to the aggregation of Pacific Islanders and Asian Americans, we cannot distill exclusive breastfeeding rates among Marshallese from other groups in the state. However, Marshallese women breastfeed at much higher rates in the Marshall Islands compared with the US, potentially suggesting that although breastfeeding is aligned with their cultural heritage, their need to work outside of the home, with a lack of workplace breastfeeding supportive policies, is a dominant barrier to breastfeeding now that they reside in the US [42,43]. It is imperative to incorporate more inclusive policies for non-professional, working women that support and encourage exclusive breastfeeding.

An unexpected barrier that emerged was a desire to formula feed to encourage independence and to foster autonomy. Encouraging independence and autonomy may be predicated on the need to work outside the home and bottle feed, creating an easier transition for others to feed the infant. The majority of the participants were young (27.1 ± 6.1) and may have exhibited a desire for autonomy and independence that older generations of Marshallese may not exhibit. Future studies should explore this desire for autonomy. This is the first study to document a desire for independence and autonomy among Pacific Islander women with regard to mother–infant attachment and infant feeding intentions.

Another unexpected finding was the preference to exclusively breastfeed females more than males. Participants described this preference as twofold. First, there was a discussion of infant-led feeding with the perception that female infants preferred breastfeeding more than male infants. Second, participants described gender breastfeeding preference deriving from a desire to not see their female infants become upset when weaning from the breast, whereas with male infants, this was not viewed the same. Previous research with Marshallese mothers identified infant- and/or child-led feeding [34,37]. However, this is the first study to identify a gender preference in infant feeding decision making among Marshallese or other Pacific Islander women.

Participants described two dominant facilitators that provided breastfeeding support and encouragement. The dominant institutional facilitator that emerged was the support from WIC. Interestingly, previous research with Marshallese mothers identified WIC as a barrier to breastfeeding, suggesting a potential change in the WIC support toward this population in the region [37]. Importantly, only 36.1% reported receiving WIC benefits. Future research should explore what specific elements of the WIC program are supportive for Marshallese mothers in their exclusive breastfeeding experiences and methods for increasing enrollment among Marshallese pregnant women.

The second facilitator was the support identified from female family members and female community members. Familial support has been identified for a myriad of reasons but specifically for health behavior decision making in other studies in the literature regarding Marshallese and other Pacific Islanders [36,60]. Future maternal education and health behavior interventions should incorporate this female-family-centric support focus.

### Limitations and Strengths

This study’s findings should be evaluated with some limitations. All participants were recruited from Arkansas, and the results may or may not be generalizable to other Pacific Islander communities residing outside Arkansas. However, establishing evidence-based interventions designed for Pacific Islanders may also inform work with other indigenous populations who have strong collectivist cultures, thus increasing the generalizability of the proposed research [61,62,63,64,65]. The qualitative methods utilized in the study allowed participants to explore the research topic in their own words. In knowing the focus of the research, participants may have tempered their responses to make them more socially acceptable. There was no control group, as this was an exploratory study. Additionally, the study design and small sample size did not allow us to draw conclusions about the prevalence of breastfeeding in Marshallese communities in Arkansas. Despite these limitations, this is the first study to document breastfeeding beliefs, influences, and intentions during pregnancy among the Marshallese population residing in the US, thus adding substantially to the literature and filling a gap in knowledge.

## 5. Conclusions

This study is the first to document the beliefs, intentions, and influences that serve as barriers and/or facilitators to exclusive breastfeeding behavior among Marshallese pregnant women residing in the US. Participants described their preferred infant feeding method to be exclusive breastfeeding, but the need to return to work was a dominant barrier, despite supportive facilitators such as female family/community members and the WIC program. These findings are being used to culturally tailor interventions to help Marshallese women achieve desired infant feeding methods.

## Figures and Tables

**Table 1:** Demographics (*n* = 36).

Response Category	N (%) or Mean ± SD
**Age**	27.1 ± 6.1
**Birthplace**	
United States	4 (11.1)
Marshall Islands	32 (88.9)
Other	0 (0)
**Duration in US (in years) (*n* = 33)**	7.8 ± 6.4
**Weeks pregnant**	32.2 ± 4.5
**Number of pregnancies**	
1 pregnancy	6 (16.7)
2 pregnancies	9 (25)
3 pregnancies	6 (16.7)
4 pregnancies	5 (13.9)
5 pregnancies	3 (8.3)
6 pregnancies	5 (13.9)
7 pregnancies	2 (5.6)
**Mean number of pregnancies**	3.4 ± 1.9
**Number of miscarriages**	
0 miscarriages	31 (86.1)
1 miscarriage	3 (8.3)
2 miscarriages	2 (5.6)
**First prenatal visit**	
First trimester	17 (47.2)
Second trimester	16 (44.4)
Third trimester	3 (8.3)
**How do you plan to feed your infant?** Exclusive breastfeeding	5 (13.9)
Breastfeeding and formula	28 (77.8)
Formula only	3 (8.3)
**Marital status**	
Single	4 (11.1)
Married	8 (22.2)
Divorced/Separated	0 (0)
Widowed	1 (2.8)
A member of an unmarried couple	23 (63.9)
**Household size**	
Number of adults	3.9 ± 1.6
Number of children	3.3 ± 1.8
Total household size	7.2 ± 2.8
**Education**	
Never attended school or only attended kindergarten	0 (0)
Grades 1 through 8 (Elementary)	3 (8.3)
Grades 9 through 11 (Some high school)	10 (27.8)
Grade 12 or GED (High school graduate)	17 (47.2)
College 1 year to 3 years (Some college or technical school)	6 (16.7)
College 4 years or more (College graduate)	0 (0)
**Employment**	
Employed for wages	0 (0)
Self-employed	3 (8.3)
Out of work for 1 year or more	10 (27.8)
Out of work for less than 1 year	17 (47.2)
Taking care of your family and home	6 (16.7)
Student	0 (0)
Retired	0 (0)
Unable to work	0 (0)
**Health insurance status**	
No	14 (38.9)
Yes	21 (58.3)
Do not know/Not sure	1 (2.8)
**Special Supplemental Nutrition Program for Women, Infants, and Children (WIC) status**	
No	23 (63.9)
Yes	13 (36.1)

**Table 2:** Qualitative themes.

Breastfeeding Beliefs and Intentions Individual Breastfeeding Beliefs Marshallese Community Breastfeeding Beliefs Infant Feeding Intentions Breastfeeding Barriers Work Independence/Autonomy Gender Breastfeeding Facilitators Institutional Facilitator Matriarchal Facilitator

## Data Availability

The de-identified data underlying the results presented in this study may be made available upon request from the corresponding author, Britni L. Ayers, at blayers@uams.edu. The data are not publicly available in accordance with funding requirements and participant privacy.

## Supplementary Materials

The The following are available online at: https://www.mdpi.com/article/10.3390/ijerph19031740/s1, Supplementary: Marshallese Mother Survey.
