# Supplementary material for: Best of Intentions: Influential Factors in Infant Feeding Intent among Marshallese Pregnant Women
Source: Int J Environ Res Public Health. 2022 Feb 3;19(3):1740. doi: 10.3390/ijerph19031740 (PMC8834797; doi:10.3390/ijerph19031740)
Supplement: Supplementary file 1 [file ijerph-19-01740-s001.zip › ijerph-1477792-supplementary.pdf]

PID Label

Marshallese Mother Survey (Pregnant Women)

Data Collector: ______________________________ **Date:** ______/______/__________

Demographic Survey

1) **What is your age? Jete am iiō?**

   ___ ___
   (Write age in years)
   If you are not sure, what do you estimate your age to be _________? Elañe kwōjjab kanooj jela, emaroñ jete am iiō?

2) Are you Marshallese? Kwe ke ri Majōl?
   ☐ Yes Aet
   ☐ No Jaab

3) **What is your Birthplace?** _________________________________ Ia eo kwar lotak ie?

   ☐ US Born Amedka
   ☐ Marshall Islands Majōl
   ☐ Don't Know/Not Sure Jab jelā/Jab kanooj jelā
   ☐ Refused Jab kōnaan uak

   How long in United States total_______________________________________**?**
   Aetokin am bed Amedka

4) **To your knowledge, are you now pregnant?** Ilo am jela, kwōj bōroro ke kiio?
   ☐ Yes Aet
   ☐ No Jaab

5) **How many weeks pregnant are you?**______________________Jete am wiik kiio?
   ☐ Don't Know/Not Sure Jab jelā/Jab kanooj jelā
   ☐ Refused Jab kōnaan uak

6) **Number of prenatal visits?________________________________** Jete aḷḷen am taktō in bōroro?
☐ Don't Know/Not Sure Jab jelā/Jab kanooj jelā
☐ Refused Jab kōnaan uak

7) **When was your first prenatal visit? ________________________** Ñaat eo jinoin tata kwar taktō in bōroro?
☐ Don't Know/Not Sure Jab jelā/Jab kanooj jelā
☐ Refused Jab kōnaan uak

8) **Number of pregnancies ____________________________** Jete kattōn am bōroro?
☐ Don't Know/Not Sure Jab jelā/Jab kanooj jelā
☐ Refused Jab kōnaan uak

9) **Number of miscarriages________________** Jete kattōn an wōr jerate ak buromōj
☐ Don't Know/Not Sure Jab jelā/Jab kanooj jelā
☐ Refused Jab kōnaan uak

10) **How do you plan to feed your infant?** Ewi wāwein am karōk ñan naajdik ajiri eo nejum?

☐ Exclusive breastfeeding Kannin wōt
If you are going to exclusively breastfeed, how long do you plan to do this ____?
Elañe kannin wōt, enaj ewi toon am naaj kōmmane wāwein in?

☐ Breastfeeding and formula Kannin im Kaninnin ilo bato
If you are going to breastfeed and use formula, how long do you plan to do this ____?
Elañe kwonaj kaninin im kaninnin ilo bato, enaj ewi toon am naaj kōmmane wāwein in?

☐ Formula only Kaninnin ilo bato wōt
If you are going to use formula only, how long do you plan to do this ____?
Elañe kwonaj kaninnin ilo bato wōt, enaj ewi toon am naaj kōmmane wāwein in?

☐ Don't know/Not sure Jab jelā/Jab kanooj jelā
☐ Refused Jab kōnaan uak

11) **Are you...?** Kwe ke juōn eo…?
- ☐ Single Ej make ian
- ☐ Married Emōj an mare
- ☐ Divorced/Separated Emōj an jebel
- ☐ Widowed Emōj an jako/mej eo ritūrūn
- ☐ A member of an unmarried couple Belele ak ejañin mare
- ☐ Refused Jab kōnaan uak

12) **Including yourself, how many people live in your home?** Ilo am kobaik kwe make, jete uwaan armij ro im rej jokwe mweo imōm?

Number of adults ________ Jete ritto
- ☐ Don't know/Not sure Jab jelā/Jab kanooj jelā
- ☐ Refused Jab kōnaan uak

Number of children ________ Jete ajiri
- ☐ Don't know/Not sure Jab jelā/Jab kanooj jelā
- ☐ Refused Jab kōnaan uak

13) **What is the highest grade you have completed?** Kilaaj jete eo iḷōñtata kwar kadedeik lok?
- ☐ Never attended school or only attended kindergarten Jañin kar jikuul ak kinter wōt
- ☐ Grades 1 through 8 (Elementary) (Kilaaj 1 ñan 8, Elementary)
- ☐ Grades 9 through 11 (Some high school) (Kilaaj 9 ñan 11, high school jidik)
- ☐ Grade 12 or GED (High school graduate) (Kilaaj 12 ak GED kadiwōjlok jen High School)
- ☐ College 1 year to 3 years (Some college or technical school) (College 1 lok ñan 3 iiō, jidik college ak ebbōk kilaaj ilo technical jikuul)
- ☐ College 4 years or more (College graduate) (College 4 iiō ak lablok, kadiwōjlok jen college)
- ☐ Don't know/Not sure Jab jelā/Jab kanooj jelā
- ☐ Refused Jab kōnaan uak

14) **Are you currently...?** Ilo tore in, kwōj ke...?
- ☐ Employed for wages Jerbal ilo jikin jerbal eo ej kōllā onem
- ☐ Self-employed Jerbal ñan kwe make
- ☐ Out of work for 1 year or more Jañin jerbal iumin 1 iiō ak lablok
- ☐ Out of work for less than 1 year Jañin jerbal 1 iiō im driklok
- ☐ Taking care of your family and home Eo ej lale ak kōkajiriri ilo kabijuknen
- ☐ A student Juōn rijikuul
- ☐ Retired Juōn eo emōj an retire
- ☐ Unable to work Jab maroñ jerbal
- ☐ Don't know/Not sure Jab jelā/Jab kanooj jelā
- ☐ Refused Jab kōnaan uak

15) **Are you a WIC recipient?** Kwōj ke bed ilo burokram in WIC eo?

☐ Yes Aet
☐ No Jaab
☐ Don't know/Not sure Jab jelā/Jab kanooj jelā
☐ Refused Jab kōnaan uak

16) **Do you have health insurance?** Ewōr ke am jabdewōt kain joortoklik/injuren in takto jen jikin wia injuren ko, einwōt HMO im injuren ko jen kien einwōt Medicare?

☐ Yes Aet
If yes, what type of insurance ____?
Elañe Aet, kain insurance rōt?
☐ No Jaab
☐ Don't know/Not sure Jab jelā/Jab kanooj jelā
☐ Refused Jab kōnaan uak
